# Adrenal and Hepatic Venous Sampling in a Case of Aldosterone-Producing Adrenocortical Carcinoma with Hepatic Metastasis

**DOI:** 10.1155/2021/5584198

**Published:** 2021-04-14

**Authors:** Toru Sugiyama, Yuriko Sasahara, Hironobu Sasano, Eri Hayakawa

**Affiliations:** ^1^Division of Endocrinology and Metabolism, Japanese Red Cross Musashino Hospital, Tokyo, Japan; ^2^Department of Pathology, Tohoku University School of Medicine, Sendai, Miyagi, Japan

## Abstract

**Background:**

Adrenocortical carcinoma (ACC) is a rare and highly aggressive malignancy. ACCs often secrete adrenal steroid hormones including cortisol and androgens; however, aldosterone-producing ACC is very rare. Although adrenal production of aldosterone is assessed by adrenal venous sampling, the use of sampling from the relevant vein to assess aldosterone production from a tumor arising from ACC metastasis has not been previously reported. *Case Presentation*. We report the case of a 69-year-old Japanese man with aldosterone-producing ACC with hepatic metastasis. He presented with a history of treatment-resistant hypertension and hypokalemia. Endocrinological examination showed markedly increased plasma aldosterone concentration and suppressed plasma renin activity. Serum cortisol concentration was not suppressed by administration of dexamethasone 1 mg, and normal circadian variation of cortisol secretion was disrupted. Abdominal computed tomography showed a large tumor in the left adrenal gland and multiple tumors in the liver. Together, these results strongly suggested ACC with multiple liver metastases causing primary aldosteronism and subclinical Cushing syndrome. Adrenal and hepatic venous sampling showed markedly increased aldosterone concentration in the left adrenal vein but no increase in the hepatic vein, despite a pathological diagnosis of ACC with hepatic metastasis, with immunohistochemical investigation showing both primary and secondary tumors to have synthetic capability for aldosterone. The patient received mitotane but declined combination chemotherapy and died 2 months later.

**Conclusion:**

This is the first report of adrenal and hepatic venous sampling in a case of aldosterone-producing ACC with hepatic metastasis. The case suggests that hepatic venous sampling is unable to detect aldosterone production from liver metastases arising from ACC.

## 1. Introduction

Adrenocortical carcinoma (ACC) is a rare, highly aggressive malignancy with an estimated annual incidence of 0.7–2.0 cases per million population. It has a heterogeneous clinical presentation, and prognosis is generally poor. Approximately 60–70% of ACCs secrete hormones, mostly glucocorticoids or androgens. Less than 10% of hormone-secreting ACCs produce aldosterone, in which cases the excessive production can cause primary aldosteronism [1–3].

Common causes of primary aldosteronism are an aldosterone-producing adenoma or bilateral idiopathic adrenal hyperplasia. The condition frequently results in difficult-to-treat arterial hypertension, sodium retention, and hypokalemia.

In cases of primary aldosteronism, adrenal venous sampling (AVS) is considered the gold standard test for determining whether the excessive aldosterone production is uni- or bilateral and identifying the adrenal gland responsible [4]. However, there have been no reports of the use of sampling from the relevant vein to assess aldosterone production from a tumor arising from ACC metastasis.

Here, we report what we believe to be the first case of aldosterone-producing ACC with hepatic metastasis in which adrenal and hepatic venous sampling were carried out to assess the production of aldosterone from the primary and secondary tumor, respectively.

## 2. Case Presentation

A 69-year-old man with an 8-month history of treatment-resistant hypertension and hypokalemia was referred to our hospital for investigation of suspected secondary hypertension. On physical examination, the only abnormal finding was hypertension (178/105 mmHg); there were no signs of Cushing syndrome (e.g., moon facies, central obesity, and buffalo hump).

Laboratory findings showed hypokalemia (2.3 mEq/L) (Table 1). The results of endocrine examination (Table 2) showed a markedly increased plasma aldosterone concentration (PAC; 1710 pg/mL) and suppressed plasma renin activity (PRA: 0.2 ng/mL/h). Serum DHEA-S (35 *μ*g/dL) was within the normal range (12–133 *μ*g/dL). Fasting plasma concentrations of the adrenocorticotropic hormone (ACTH) and serum cortisol were 2.1 pg/mL (below the lower limit of normal, 7.2 pg/mL) and 9.4 *μ*g/dL (within the normal range, 4.0–18.2 *μ*g/dL), respectively. Serum cortisol concentration was not suppressed in response to an overnight low-dose (1 mg) dexamethasone suppression test, and normal circadian variation of cortisol secretion was disrupted. Both urinary aldosterone concentration and cortisol concentration were increased: 549 *μ*g/24 h (normal, <10 *μ*g/24 h) and 328 *μ*g/24 h (upper limit of normal, 80.3 *μ*g/24 h), respectively.

**Figure 1:**
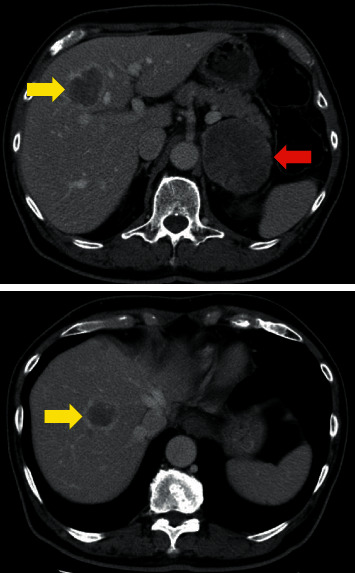
Abdominal computed tomography scan. The red arrow indicates the left adrenal tumor (diameter, 7 cm). The yellow arrows indicate multiple liver tumors (diameter, ∼4 cm).

Abdominal computed tomography (CT) showed a left adrenal tumor (diameter, 7 cm) and multiple liver tumors (diameter, ∼4 cm) (Figure 1).

The combined examination and investigation results strongly suggested ACC with multiple liver metastases causing primary aldosteronism and subclinical Cushing syndrome. Such cases are classified as clinical stage IV and therefore ineligible for surgery to remove the adrenal tumor. However, the patient hoped to have the adrenal tumor removed, so he requested investigation of the liver tumors to confirm that they had arisen from the ACC.

**Figure 2:**
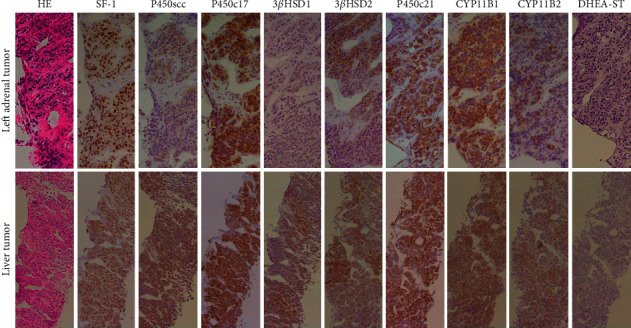
Results of histopathological and immunohistochemical investigation of left adrenal and liver biopsy specimens (magnification, ×200). Microscopic appearance (hematoxylin and eosin, HE, staining) showed eosinophilic tumor cell cytoplasm, nuclear atypia, diffuse architecture, and sinusoidal invasion. Immunohistochemical staining showed strong positive reactivity for SF-1, P450c17, 3*β*HSD2, P450c21, CYP11B1, and CYP11B2 and partial positive reactivity for P450scc and 3*β*HSD1 in both the left adrenal tumor (upper panel) and the liver tumor (lower panel). The DHEA-ST test result was negative for both specimens.

Adrenal and hepatic venous sampling were carried out. Aldosterone concentration was markedly increased in the left adrenal vein (64,500 *μ*g/dL) but not in the right adrenal or hepatic vein (4870 and 1210 *μ*g/dL, respectively) (Table 3). These findings suggested that the liver tumors were not secreting aldosterone; therefore, biopsies of tissue from both the left adrenal tumor and the liver tumors were carried out to enable pathological diagnosis. Based on the Weiss criteria, the biopsy results for the left adrenal and liver tumors were pathologically consistent with the diagnosis of ACC with hepatic metastasis. Immunohistochemical investigation showed strong positive staining for SF-1, P450c17, P450c21, 3*β*HSD2, CYP11B1, and CYP11B2 and partial positive staining for P450scc and 3*β*HSD1 in both the ACC and the liver metastases (Figure 2); these findings indicated synthetic capability for aldosterone and cortisol. The DHEA-ST test result was negative for both the ACC and the liver metastases.

The patient received mitotane (1500 mg/day) but declined combination chemotherapy. Hypoalbuminemia and severe edema of the lung and lower extremities developed rapidly despite administration of high doses of spironolactone, and he died 2 months later.

## 3. Discussion

To our knowledge, this is the first report of a case of aldosterone-producing ACC with hepatic metastasis in which adrenal and hepatic venous sampling were carried out to assess the production of aldosterone from metastasis of ACC. This was an unusual case because hypersecretion of aldosterone from ACC is rare; in the majority of cases (approximately 60–70%), ACCs present with an excess of adrenal hormone (mostly glucocorticoids or androgens) [1–3].

Although AVS is considered the gold standard test for determining whether the right, the left, or both adrenal glands are responsible for the aldosterone excess in cases of primary aldosteronism, to our knowledge, there have been no reports of the use of sampling from the relevant vein to assess aldosterone production from a tumor suspected to have arisen from ACC metastasis. In the case presented in this report, we used hepatic venous sampling to try to help determine whether the liver tumors were ACC metastases or other hepatic tumors (e.g., hepatocellular carcinoma). However, aldosterone concentration in the hepatic veins was not increased, despite the pathological diagnosis of hepatic metastasis of ACC and its synthetic capability for aldosterone.

In cases of insulinoma, hepatic venous sampling after selective intra-arterial injections of calcium into the relevant hepatic artery is carried out to exclude hepatic metastasis of insulinoma [5–8]. In a similar way, liver metastases arising from ACC might be expected to be detected by hepatic venous sampling. Because aldosterone is a lipophilic steroid hormone, it should be released into the blood circulation once synthesized. Our experience in this case suggests that hepatic venous sampling is unsuitable for diagnosis of ACC with hepatic metastasis. Although the liver tumors were found by immunohistochemical investigation to have synthetic capability for aldosterone and cortisol, aldosterone concentration in the hepatic veins was found not to be increased. This might be explained by the limited transportation of aldosterone to the hepatic vein or degradation of aldosterone during its passage through the liver. Further basic research is required to explain this finding.

Adrenocortical carcinoma has a generally poor prognosis, with overall 5-year survival ranging from 60–80% in patients with stage I disease to 13% in those with stage IV. Clinical outcomes vary greatly due to differences in tumor biology, disease presentation, and management options [1, 2, 9, 10]. The results of a recent systematic review and meta-analysis show that cortisol-secreting ACCs are associated with worse overall survival [11]. This higher mortality rate could be explained by negative effects of cortisol on the immune and cardiovascular systems. In cases of aldosterone-secreting ACCs, scarcity of data means that prognosis is uncertain. In the case presented here, the marked hyperaldosteronism could have negatively affected the cardiovascular system and electrolyte balance and thus contributed to the poor outcome.

Management of ACC remains challenging due to the heterogeneous and often unpredictable nature of this disease. If our patient's liver metastases had not produced excessive aldosterone, surgical resection of the primary tumor might have resolved the hyperaldosteronism, and this might have led to a better outcome.

## 4. Conclusion

We report the first case of adrenal and hepatic venous sampling in a patient with aldosterone-producing ACC with hepatic metastasis. Hepatic venous sampling did not detect aldosterone production from the liver tumors.

## Figures and Tables

**Table 1:** Laboratory findings.

Blood cell count	Biochemistry				
WBC (/*μ*L)	6000	TP (g/dL)	7.0	UN (mg/dL)	11.5
Neutrophil (%)	62.2	Alb (g/dL)	4.2	Cr (mg/dL)	0.59
Lymphocyte (%)	28.3	LDH (IU/L)	330	UA (mg/dL)	4.1
Monocyte (%)	8.3	AST (IU/L)	22	Na (mEq/L)	147
Eosinophil (%)	0.7	ALT (IU/L)	39	K (mEq/L)	2.3
Basophil (%)	0.5	*γ*-GT (IU/L)	31	Cl (mEq/L)	95
RBC (× 10^6^/*μ*L)	4.47	T-Bil (mg/dL)	0.8	Ca (mg/dL)	8.7
Hemoglobin (g/dL)	13.0	ALP (IU/L)	308	P (mg/dL)	2.6
Hematocrit (%)	38.2	Plasma glucose (mg/dL)	95	T-chol (mg/dL)	167
Platelet (× 10^4^/*μ*L)	24.5	CK (IU/L)	216	HDL-c (mg/dL)	60.7
		CRP (mg/dL)	0.17	LDL-c (mg/dL)	95.6
				TG (mg/dL)	95

**Table 2:** Endocrinological examination.

Hormonal profile			Normal range	
Plasma ACTH (pg/mL)	2.1	7.2–63.3		
Serum cortisol (*μ*g/dL)	9.4	4.0–18.3		
Plasma renin activity (ng/mL/h)	0.2	0.3–2.9		
PAC (pg/mL)	1710	140–1030		
Serum DHEA-S (*μ*g/dL)	35	12–133		
Urinary free cortisol (*μ*g/24 h)	328	11.2–80.3		
Urinary free aldosterone (*μ*g/24 h)	549	<10		
Circadian variation of plasma ACTH and serum cortisol and low-dose (1 mg) DST results				
Time	06:00	16:00	23:00	1 mg DST
Plasma ACTH (pg/mL)	<1.0	<1.0	<1.0	<1.0
Serum cortisol (*μ*g/dL)	9.8	11.3	11.6	12.0

**Table 3:** Adrenal and hepatic venous sampling.

	Sampling point	PAC (pg/mL)	Cortisol (*μ*g/dL)	PAC : cortisol ratio
Before ACTH loading	Inferior vena cava	3890	18.3	21.3 × 10^−3^
	Left adrenal vein	35,000	44.7	78.3 × 10^−3^
	Right adrenal vein	3310	23.2	14.3 × 10^−3^
	Hepatic vein	1360	18.8	7.2 × 10^−3^

After ACTH loading	Inferior vena cava	4260	21.4	19.9 × 10^−3^
	Left adrenal vein	64,500	58.2	110.8 × 10^−3^
	Right adrenal vein	4870	132	3.7 × 10^−3^
	Hepatic vein	1210	21.2	5.7 × 10^−3^

## Data Availability

The datasets used and/or analyzed during the current study are available from the corresponding author upon reasonable request.

## Abbreviations

3*β*HSD: 3*β*-Hydroxysteroid dehydrogenase
ACC: Adrenocortical carcinoma
ACTH: Adrenocorticotropic hormone
Alb: Albumin
ALP: Alkaline phosphatase
ALT: Alanine aminotransferase
AST: Aspartate aminotransferase
CK: Creatine kinase
Cr: Creatinine
CRP: C-reactive protein
CYP: Cytochrome P450
DHEA-ST: Dehydroepiandrosterone sulfotransferase
DST: Dexamethasone suppression test
HDL-c: High-density lipoprotein cholesterol
HE: Hematoxylin and eosin
LDH: Lactase dehydrogenase
LDL-c: Low-density lipoprotein cholesterol
P450c17: Cytochrome P450 17
P450c21: Cytochrome P450 21
P450scc: Cytochrome P450 side-chain cleavage
PAC: Plasma aldosterone concentration
RBC: Red blood cell
SF-1: Steroidogenic factor 1
T-Bil: Total bilirubin
T-chol: Total cholesterol
TG: Triglycerides
TP: Total protein
UA: Uric acid
UN: Urea nitrogen
WBC: White blood cell
*γ*-GT: *γ*-Glutamyltranspeptidase.
